# Perceptions about sexual risk, HIV and HIV-testing in Cali, Colombia

**DOI:** 10.25100/cm.v49i2.2945

**Published:** 2018-06-30

**Authors:** Héctor Fabio Mueses-Marín, Inés Constanza Tello-Bolívar, María Isabel Galindo-Orrego, Jaime Galindo-Quintero

**Affiliations:** Grupo Educación y Salud en VIH/Sida. Corporación de Lucha Contra el Sida, Cali, Colombia.

**Keywords:** HIV, Colombia, risky behavior, unsafe sex, Screening, VIH, Colombia, conducta de riesgo, sexo inseguro, Tamizaje.

## Abstract

**Introduction:**

In Colombia, 20%-54% of the population with sexual practices at higher risk for HIV infection (men who have sex with men, transgender women, women sex workers) has sometime been tested.

**Objective:**

To describe perceptions of sexual risk, HIV and HIV testing in people with risky sexual practices and people who identify themselves as heterosexual.

**Methods:**

Between 2012 and 2014, it was carried a descriptive study using HIV screening out in Cali-Colombia with the voluntary participation of 940 people aged over 18 years. There were used: informed consent, structured questionnaire and HIV testing. Descriptive, bivariate and multivariate Poisson regression models were performed.

**Results:**

Average age 28.5 ±10.9 years; 50% men. 357 (38%) were people from the traditional risk group for HIV infection; and 583 (62%) corresponded to the non-traditional risk group (heterosexual men and women). Likewise, 62% and 41% respectively had HIV test. 51% to 53% reported having sex when they consumed liquor; commercial sex was higher in the group with risky sexual practices (32% vs 3%), as well as anal relations (77% vs 23%), consistent use of condom (32% vs 9%), and HIV positive test (14.3% vs 1.6%). The multivariate analysis showed for both groups that having HIV test was associated with being older than 25 years and a history of sexually transmitted infection.

**Conclusions:**

Differentiated education strategies are needed based on risky sexual behaviors and that consider the importance of regular HIV tests for early diagnosis and timely enrollment in care and treatment.

## Resumen

**Introducción:**

En Colombia, 20%-54% de la población con prácticas sexuales de mayor riesgo para infección por VIH (Hombres que tienen sexo con hombres, mujeres transgénero, mujeres trabajadoras sexuales) se ha hecho la prueba alguna vez.

**Objetivo:**

Describir las percepciones sobre riesgo sexual, VIH y prueba del VIH, en personas con prácticas sexuales de riesgo y personas que se identifican heterosexuales.

**Métodos:**

Entre 2012 y 2014 se hizo en Cali-Colombia un estudio descriptivo mediante tamizaje para VIH con participación voluntaria de 940 personas mayores de 18 años. Fueron utilizados: consentimiento informado, cuestionario estructurado y prueba VIH. Se realizaron análisis descriptivos, bivariado y modelo de regresión multivariada Poisson.

**Resultados:**

Edad promedio 28.5 ±10.9 años, 50% hombres. 357(38%) eran personas de grupos tradicionales de riesgo para infección VIH y 583(62%) correspondían a grupo no tradicional de riesgo (hombres y mujeres heterosexuales). Así mismo, 62% y 41% respectivamente, tenían prueba de VIH. Reportaron tener relaciones sexuales cuando consumieron licor 51% a 53%; sexo comercial fue mayor en grupo con prácticas sexuales de riesgo (32% vs 3%), igual que relaciones anales (77% vs 23%), uso consistente de condón (32% vs 9%) y prueba VIH positiva (14.3% vs 1.6%). El análisis multivariado mostro para ambos grupos, que tener prueba de VIH se asoció con ser mayor de 25 años y antecedente de infección de transmisión sexual.

**Conclusiones:**

Se necesitan estrategias de educación diferenciadas según comportamientos sexuales de riesgo y que consideren la importancia de las pruebas regulares del VIH para el diagnóstico temprano e inscripción oportuna en la atención y tratamiento.

## Introduction

Up to 2015, there were notified 112,110 HIV-positive or AIDS-positive persons in Colombia, and 12,764 deceased persons ^1^. According to data from the Ministry of Health and Social Protection of Colombia, the prevalence of HIV infection in the population aged 15 to 49 years in 2013 was around 0.5% ^2^ .

In Colombia, voluntary HIV testing is close to 20% in the general population ^3^, and it ranged from 20% to 54% in populations at highest risk for infection ^4^ ^,^ ^5^; for the year 2012, it was reported that only 18.4% of people diagnosed with HIV/AIDS in Colombia had requested voluntary performance of the test for diagnosis ^6^. Studies of HIV infection in populations traditionally considered at risk (men who have sex with men (MSM), transgender women (TRANS) and women sex workers (WSW)) showed HIV prevalence values for the city of Cali ranging from 1.7% in women sex workers, up to 23.7% in men who have sex with men ^4^ ^,^ ^7^ ^,^ ^8^. In parallel, the HIV test history statistics in high risk populations was close to 63% ^7^ ^,^ ^8^. Reports for general population in the city of Cali, in a context of low economic level, presented HIV+ frequencies close to 2% ^9^, and HIV test history ranging from 30% to 36% ^9^ ^,^ ^10^.

The scientific literature on the subject indicates that the majority of people infected with HIV do not take the test before reaching advanced stages of infection ^11^. Added to this, a large number of infected people cannot access the diagnosis for different reasons, among which are the fear of stigma and discrimination, the cost of the test, or the resistance to the recognition that they are at risk ^12^ ^-^ ^15^.

For the city of Cali, it was expected for 2015 an increase of between 10-20% ^16^ in the goals that strengthen the demand and offer of counseling and voluntary testing of sexually transmitted infections-HIV/AIDS, for an established baseline for the city that oscillated between 20% and 32% in the traditional group of risk (MSM, TRANS and WSW). Despite the efforts in recent years to improve access to testing, especially in people with limited resources or those who are part of traditional risk group for infection, even prevention plans and services for infection are limited or ineffective ^5^.

In the national context, information continues to be scarce compared to the characterization of people who go to tests in order to know their HIV serum status, as well as knowledge of statistics on HIV testing. At global and country level, there is a consensus on the importance of testing for HIV. In parallel, the fact that the administration of antiretroviral therapy in people with HIV results in a significant reduction in the transmission of HIV ^17^ encourages an increase in the diagnosis of the infection, with the purpose of improving the opportunity to enter the cascade of HIV, attention and treatment. The present study investigated perceptions of sexual risk, HIV and HIV testing, as well as factors related to the HIV test history in people belonging to traditional risk group for HIV infection, together with couples of people with HIV/AIDS and people who identify with heterosexuals, who received counseling and voluntary testing for the prevention of HIV infection.

## Materials and Methods

### Design

Observational cross-sectional study, whose results are part of a sub-analysis of data collected between 2012 and 2014 in Cali-Colombia, during the implementation of an active HIV search strategy ^10^ that included counseling and HIV testing. 

### Context of the study

The study was carried out in the city of Cali, located in the southwest of Colombia, with a total population of approximately 2.3 million people. It was estimated that 38% of the population of Cali is affiliated with the subsidized health system (Datos Cali en cifras 2014, Alcaldia Santiago de Cali. https://planeacion.cali.gov.co/caliencifras/Documentos%20pdf/Caliencifras2014.pdf),, which in turn indicates that these people belong to the lowest socioeconomic level in the country.

### Target population and sample size

By non-probabilistic sampling, there participated voluntarily people over 18 years of age, of both sexes, of low socioeconomic status. The sample consisted of two groups: 1) people from the traditional risk group (MSM, TRANS and WSW) and couples of people with HIV/AIDS (CPH); 2) people who at the time of the interview were recognized as heterosexual and not belonging to the traditional risk group. All the participants were from low socio-economic strata 1 and 2, established according to the classification of their public utilities receipts for water and energy in the city (data that was self-reported by the participants).

### Recruitment strategy

The invitation to participate was made through community leaders belonging to interest groups, who served as liaison to inform about a local center where participants could receive counseling and access to HIV testing. The search and development activities of the study were carried out in community centers, places of sexual work, and in the facilities of an ambulatory care center for people living with HIV. The interview and counseling was carried out by personnel of the research team (physician, nurse and social worker) as part of comprehensive focal active search campaigns for counseling and voluntary HIV testing (CVT), according to national guidelines ^18^.

### Collection of information

It was designed a structured questionnaire (SQ) by the research team, in order to obtain information on socio-demographic characteristics, self-perception of knowledge about risk and HIV prevention, as well as variables related to sexual behaviors and a history of sexually transmitted infections (STI); these questions took into account the guidelines of the questionnaires of the HIV/AIDS prevention guidelines of the United Nations Population Fund for Colombia ^8^ ^,^ ^19^. The content of the questionnaire was reviewed by a research committee with experience in HIV, and adjusted according to the results of a pilot study. It was carried out a face-to-face interview by a trained professional (nurse, social worker or general practitioner), in parallel with counseling for the HIV test, which allowed the immediate clarification of doubts or false beliefs recorded in the questionnaire. The SQ was administered on paper and in a private place.

### Study variables

#### HIV test history

The outcome variable of the study corresponded to having a previous test for HIV infection at the time of participation. To evaluate the HIV test history, each participant was asked the following: if they had ever had an HIV or AIDS test; later, for those who gave a positive response, it was inquired if the test was done on their own initiative; likewise they were asked in which month and year the test was performed for the last time, without including blood donations.

#### Independent variables

**Socio-demographic aspects**. Through the questionnaire, there were identified age, sex, educational level, race, socioeconomic stratum, marital status, health insurance affiliation and monthly income.

**Use of alcohol and drugs**. It was inquired about the consumption of alcohol and use of psychoactive substances (PS).

**Tattoos and/or piercing**. It was investigated if they had gotten any tattoo or piercing during the last year.

**Sexual behavior**. Risk behaviors related to HIV were evaluated, including knowledge about the transmission routes (which was calculated based on the percentage of correct responses related to HIV transmission routes), knowing someone with HIV, living together at the current time or suspecting that one of their partners had HIV. We also inquired about portability and condom use with their partners, anal/oral intercourse practices, number of sexual partners (regular, occasional or commercial), sex for money/benefits, history of STI diagnosis by a doctor or health professional. 

### HIV diagnosis

For the diagnosis of HIV, it was used the diagnostic algorithm according to the national guideline ^20^, which was in effect during the study period. A first sample was taken and evaluated with a qualitative DoubleCheckGold™ HIV 1&2 rapid immunoassay test (sensitivity and specificity of 99.9% and 99.6% -99.8%, respectively) ^21^. If the first sample was reactive, a second sample was made on filter paper, to which a fourth-generation Elisa test was performed. If this test was reactive, a Western Blot test was carried out for confirmation in the same reference laboratory; if Elisa was non-reactive, the rapid screening test was considered a "false positive".

### Statistical analysis

The study groups were initially described by frequencies and proportions. Group comparisons were made through the Pearson Chi square test and trend in the variables with more than two categories, whose results are illustrated in Tables 1 and 2.

On order to estimate the possible relationship between the HIV test history with the socio-demographic characteristics, knowledge about the risk and prevention of HIV, sexual behaviors and STI antecedents, there were calculated and modeled prevalence ratios within each group, which were estimated using a multivariate Poisson regression model with robust variance ^22^ ^,^ ^23^. (Results presented in Tables 3 and 4). All statistical analyses were performed with Stata intercooler® version 12. The level of significance used for the final model was 0.05.

### Ethical aspects

The study was carried out in accordance with the ethical principles related to human experimentation established in Colombia by Resolutions 8430 of 1993 and 2378 of 2008, as well as the "Declaration of Helsinki" and its amendments. Participation in the study was completely voluntary, and an informed written consent was obtained from each participant, both for counseling and taking HIV test and for the structured questionnaire administered in the study. This study was reviewed and approved by the institutional ethics committee for human research of the Corporation to fight AIDS (Cali-Colombia).

## Results

### Descriptive

A total of 940 people participated. The average overall age of the participants was 28.5 ± 10.9 years; half of them were men.

### Analysis by groups

Both groups had an average age of 28 years. The detailed information on the general characteristics of the participants in each group is illustrated in Table 1.

**Table 1:** Comparison of socio-demographic characteristics, behaviors and antecedents, according to study groups. Cali-Colombia, 2012-2.

	Traditional risk group (MSM, TRANS, WSW and CPH)^*^ n= 357		Non-traditional risk group (heterosexual men and women)^*^ n= 583		P^**^
	n	%	n	%	
Age (years)					
18-25	173	49	318	54	<0.001
26-35	105	29	121	21	
36-45	58	16	75	13	
<45	21	6	69	12	
Sex					
Male	255	71	216	37	<0.001
Female	102	29	366	63	
Description of traditional risk group					
Men who have sex with men	203	57			
Transgender women	35	10			
Women sex workers	89	25			
Couples of people with HIV/AIDS	30	8			
School level					
Primary school			50		14	113	19	0.005
Secondary school		207		58		351	61	
Technical-superior		100		28		115	20	
Civil status					
Married - Free Union	88	25	246	42	<0.001
Single-Separated-Widow	266	75	334	58	
Health insurance/coverage					
Yes	281	80	526	92	<0.001
Socioeconomic stratum					
One (very low)	165	46	328	56	0.003
Two (low)	192	54	254	44	
Current monthly income (SMML)					
No income	118	33	309	54	<0.001
<1	122	34	166	29	
1-5	115	33	94	17	
Liquor consumption during the last 30 days					
Yes	209	63	286	50	<0.001
Sexual intercourse when consuming liquor					
Yes	164	51	288	53	0.677
PS consumption					
Yes	183	52	218	38	<0.001
Tattoos and/or piercings during the last year					
Yes	78	22	85	15	0.006
Being diagnosed with an STI by a physician					
Yes	70	20	80	14	0.017
Knowing anybody with HIV or who died from AIDS					
Yes	196	55	211	36	<0.001
Living with a person with HIV/AIDS					
Yes	66	19	6	1	<0.001
Knowing or suspecting that the current/past couple(s) has/had VIH					
Yes	118	33	46	8	<0.001
(Knowing that) People can get protected by using a condom in their sexual relations					
Yes	305	85	515	88	0.172
Knowing about HIV infection					
Adequate	74	21	113	20	0.917
Having intercourse within the last 12 months					
Yes	344	97	523	90	<0.001
Having anal intercourse					
Yes	269	77	125	23	<0.001
Having oral intercourse					
Yes	303	85	324	56	<0.001
Getting money/benefits in exchange for sex within the last 12 months					
Yes	115	32	18	3	<0.001
Using condom within last 12 months-All couples					
Yes, always	115	32	50	9	<0.001
Carry condoms					
Yes	235	66	217	37	<0.001
Test history for HIV					
Yes	223	62	240	41	<0.001
Time since last HIV test performed (year)					
<1	79	37	33	16	
1-3	78	37	79	38	
3-5	23	11	43	20	
>5	33	15	55	26	
Test conducted on own initiative (yes)	180	83	158	67	<0.001
Frequency of result HIV+	51	14.3	9	1.6	

* Calculation of column percentages

** p-value obtained from the chi-2 test

SMML: Spanish acronym for monthly minimum wage

### Test history for HIV

People who shared characteristics of traditional risk group had a higher frequency of HIV test history. Likewise, the frequency of positivity for HIV was higher in this group (Table 1).

Considering the differences found between the groups (Table 1), a separate analysis was carried out of the general characteristics, behaviors and knowledge in relation to the antecedent of HIV tests, which are presented in Table 2.

**Table 2:** General characteristics of behaviors and knowledge evaluated in the study groups, according to antecedent of test for HIV (variables with significance <0.15 in at least one of the groups). Cali-Colombia, 2012-2014

	Traditional risk group (MSM, TRANS, WSW and CPH)^*^					Non-traditional risk group (heterosexual men and women)^*^				
	With previous test (n= 223)		Without previous test (n= 134)		p**	With previous test (n= 240)		Without previous test (n= 343)		p**		n	%	n	%		n	%	n	%	
Age (years)										
18-25	76	34	97	73	<0.0001	110	46	207	60	<0.0001
26-35	82	37	23	17		78	32	43	13	
≥36	65	29	14	10		52	22	92	27	
Sex										
Male	145	65	110	82	<0.001	65	27	151	44	<0.001
Current civil status										
Married - Free Union	57	26	31	23	0.645	131	55	115	34	<0.001
Single	165	74	101	77		109	45	225	66	
Current monthly income (SMML)										
No incomes	50	23	68	51	<0.001	127	54	182	54	0.958
<1	87	39	35	26		70	30	96	29	
1-5	85	38	30	23		38	16	56	17	
Liquor consumption during the last 30 days										
Yes	138	66	71	57	0.097	130	55	156	47	0.055
Sexual intercourse when consuming liquor										
Yes	114	57	50	41	0.006	108	47	152	48	0.846
PS consumption										
Yes	126	58	57	44	0.013	96	40	122	36	0.327
Liquor and PS										
Yes	29	13	8	6	0.038	8	3	6	2	0.228
Tattoos and/or piercings during the last year										
Yes	50	23	28	21	0.746	24	10	61	18	0.01
Being diagnosed with an STI by a physician										
Yes	60	27	10	8	<0.001	49	61	31	39	<0.001
Knowing anybody with HIV or who died from AIDS										
Yes	139	63	57	43	<0.001	91	38	120	35	0.486
Knowing or suspecting that the current/past couple(s) has/had VIH										
Yes	81	37	37	28	0.075	19	8	27	8	0.982
Having anal intercourse										
Yes	161	74	108	83	0.040	55	24	70	22	0.573
Having oral intercourse										
Yes	185	84	118	91	0.093	140	62	184	59	0.433
Getting money/benefits in exchange for sex within the last 12 months										
Yes	92	50	23	21	<0.001	11	5	7	2	0.097
Carry condoms										
Yes	162	74	73	55	<0.001	89	39	128	39	0.931

* Percentages calculation row;

** p-value obtained from the chi-2 test

SMML: Spanish acronym for monthly minimum wage

### General characteristics of the study participants and their relation to a previous test history for HIV 

#### Multivariate Analysis

For each group, there were entered the variables with levels of significance less than 0.15 into a Poisson multiple regression model (Table 3), from which it was obtained that, in both groups, characteristics such as age and the antecedent of medical diagnosis for some STI were associated with a history of HIV testing. In the traditional risk group, commercial sex practices within the last twelve months and suspecting that the current partner(s) had HIV were related to having a prior history for HIV. In the case of people not belonging to the traditional risk group, it was observed that being a woman and living together as a couple were the most frequent characteristics in those who reported the antecedent of getting an HIV test (Table 3).

**Table 3:** Factors related to antecedent of getting an HIV test, according to groups in Cali-Colombia, 2012-2014

	Antecedent of getting an HIV test							
	Traditional risk group (MSM, TRANS, WSW and CPH)				Non-traditional risk group (heterosexual men and women)			
	PR	SE	p	95% CI	PR	SE	p	95% CI
Age (years)								
18-25	Reference				Reference			
26-35	1.51	0.16	<0.001	1.23-1.85	1.44	0.16	0.001	1.16-1.79
Over 35	1.68	0.20	<0.001	1.33-2.12	0.75	0.12	0.069	0.56-1.02
Sex								
Male	Reference				Reference			
Female	1.04	0.13	0.728	0.82-1.32	1.50	0.19	0.001		1.18-1.92
Civil status								
Married - Free Union	Reference				Reference			
Single	0.90	0.08	0.252	0.75-1.08	0.65	0.07	<0.001		0.53-0.81
Being diagnosed with an STI by a physician								
No	Reference				Reference			
Yes	1.25	0.11	0.007	1.06-1.48	1.72	0.18	<0.001		1.40-2.10
Knowing or suspecting that the current/past couple(s) has/had VIH								
No	Reference				Reference			
Yes	1.27	0.14	0.023	1.03-1.57	0.92	0.19	0.671		0.61-1.37
Getting money within the last 12 months in exchange for sex								
No	Reference				Reference			
Yes	1.52	0.17	<0.001	1.23-1.88	1.14	0.12	0.215	0.92-1.41

RP: Prevalence ratio obtained from the Poisson multivariate model;

SE: standard error calculated by robust methods;

*p*: *p* value obtained for each RP estimator of the multivariate Poisson model;

CI 95%: 95% confidence interval obtained for each RP estimator of the multivariate Poisson model

Table 4 shows the results for both groups when the multivariate analysis was considered by the antecedent variable of getting an HIV test on the participant's own initiative.

**Table 4:** Factors related to antecedent of performing a test for HIV at the initiative of the participant, according to groups. Cali-Colombia, 2012-2014

	Antecedent of performing a test for HIV at the initiative of the participant							
	Traditional risk group (MSM, TRANS, WSW and CPH)				Non-traditional risk group (heterosexual men and women)			

Age (year)										
18-25	Reference				Reference			
26-35	1.80	0.28	<0.001	1.33-2.43	1.70	0.31	0.003	1.20-2.42
Over 35	2.24	0.35	<0.001	1.65-3.05	1.04	0.22	0.850	0.68-1.58
Civil status										
Married - Free Union	Reference				Reference			
Single	0.89	0.11	0.346	0.70-1.13	0.72	0.11	0.036	0.53-0.98
Knowing or suspecting that the current/past couple(s) has/had VIH										
No	Reference				Reference			
Yes	1.36	0.18	0.020	1.05-1.75	1.20	0.32	0.495	0.71-2.03
Getting money within the last 12 months in exchange for sex										
No	Reference				Reference			
Yes	1.31	0.16	0.031	1.02-1.67	1.62	0.38	0.038	1.03-2.56
Using condom within last 12 months-All couples										
Not always	Reference				Reference			
Always	1.40	0.16	0.003	1.12-1.74	0.96	0.32	0.909	0.50-1.8
Carry condoms										
No	Reference				Reference			
Yes	1.36	0.20	0.043	1.01-1.82	1.16	0.19	0.367	0.84-1.59

PR: Prevalence ratio obtained from the Poisson multivariate model;

SE standard error calculated by robust methods;

*p*: *p*-value obtained for each RP estimator of the multivariate Poisson model;

95% CI: 95% confidence interval obtained for each RP estimator of the multivariate Poisson model

The variables associated with the HIV test history on own initiative in the people of the traditional group of risk were age over 25 years, commercial sex behaviors and condom use. While for the non-traditional group of people at risk, the antecedent of getting an HIV test on their own initiative was associated with the category of the age range of 26-35 years, being single and commercial sex (Table 4).

## Discussion

The frequency of antecedent test was close to 50% in the total sample, and it was mainly related to variables such as age, sex, marital status, history of STI, suspicion of a couple with HIV, commercial sex and condom use. However, people from the traditional risk group reported higher frequency of previous test (62%). The results suggest that at least one third of the population in the present study had never been tested for HIV. In Colombia, HIV testing is voluntary, not mandatory, and must always be preceded by counseling, and it is covered by the Compulsory Health Plan. However, in traditional populations at risk within the national guidelines, there is no evidence of any recommendation for the frequency of completion of the HIV test ^18^. In this regard, the US Center for Disease Control and Prevention recommends that all people aged between 13 and 64 years be tested for HIV at least once, as part of their routine medical care; and people with certain factors risk should be tested more frequently, at least once a year ^24^.

The frequency of positivity for HIV remains high in people in a context of higher risk (Table 1), similar to that reported for Colombia in these populations ^4^ ^,^ ^7^ ^,^ ^8^. For the non-traditional group of people at risk, the positivity for HIV was higher than the national estimate in the general population ^2^. These results demonstrate the need to guide differentiated strategies in the populations that promote continuity in HIV prevention efforts that have been carried out in the country.

Different studies ^3^ ^,^ ^9^ ^,^ ^25^ ^-^ ^27^ have shown that demographic factors contribute to the explanation of performing HIV tests. In agreement with the above, in the present study, age was the demographic factor that was related to having previous test in both groups; and sex (being female), mainly for the non-traditional risk group, it was found that people older than 26 years of the traditional risk group were more likely to present a history of being tested, which was similar with people in the age range of 26 to 35 years belonging to the non-traditional risk group, but they behaved inversely in those aged over 35 years within the same group. In this regard, a study in the general population of Colombia reported that people aged over 65 are less likely to have an HIV test ^3^. The results suggest that more efforts should be made, differentiated by age and by risky sexual behavior that people share, as a guide to optimize the promotion of HIV testing.

The antecedent report of STI was related to having a previous test in both groups, observing that between 61% and 86% of those who reported having an STI diagnosis had a previous HIV test, which suggests the need to expand the opportunities of the HIV test in STI care centers and in clinical settings similar to those for attending people with higher sexual risk, as well as the adult population in general.

Suspicion or knowledge that the current or past partner was HIV+, was associated with having a previous test for the diagnosis of HIV infection, only in the traditional risk group; which may suggest that perceiving this type of risk in the couple favors the performance of a test for HIV; however, the temporality of the study limits us in being able to confirm this statement, since we did not investigate if the test was preceded by the suspicion of HIV in the couple or another reason related to their risk behaviors in themselves, which has been evidenced in MSM who have been previously tested for HIV, in whom there have been found a higher probability of reporting risk behaviors for HIV associated with performing the test ^28^. 

The experience of a sexual relationship (in exchange) for money was associated with a higher frequency of the antecedent of the HIV test, for both groups, mainly when the previous test was on the initiative of the participant. Several studies have reported that those who share risky behaviors for HIV infection tend to have more antecedents of tests for HIV ^28^; in some cases, this could be motivated by the related belief that commercial sex work is highly risky for the transmission of HIV ^29^ ^,^ ^30^. The results highlight the importance of proposing education strategies in people who participate in activities related to commercial sex, which allow greater access to HIV test and that are consistent with the frequency of performing it, as it has been suggested for this type of population ^24^. 

The evaluation of associations found in both groups, considering the antecedent of taking the test (Table 3), as well as the antecedent of doing it at the initiative of the participant (Table 4), include variables that suggest risk behaviors, specifically in the non-traditional risk group, in which a relationship was observed between taking a test on their own initiative and having sex for money. In addition, when assessing the presence of antecedents of having the test on their own initiative in the traditional risk group, it was (always) reported a greater use of condom with all couples, as well as the fact of carrying condoms; this last fact could suggest a greater awareness of protected sex practices in this group, although the literature is not clear about condom use and HIV test history ^31^ ^-^ ^33^. The findings seem to suggest that when people experience risk behaviors for HIV infection, they are motivated to correct their behaviors and/or to take an HIV test. It is necessary to promote access to the test through the spontaneous demand of the population, as well as strategies that eliminate the barriers and the stigma against the (HIV) test.

The people of the non-traditional group of risk were mostly women; in this sense, the results suggest that those who take the test the most are mainly women of childbearing age and married or in free union; this relationship could be mediated by the presence or desire for pregnancy (data not evaluated in the present study); in this regard, the country's policy establishes that health centers and hospitals with prenatal care programs are obliged to offer HIV testing to every pregnant woman ^34^, which can explain in some way why the antecedent of HIV test is more frequent in women within this group. These results suggest the need to encourage more the promotion of HIV testing in men who are considered heterosexual and not considered within the traditional risk groups, as well as to take advantage of prenatal care programs, in order to suggest HIV testing to men and not only to pregnant women.

The cross-sectional design of this study limits our ability to evaluate temporal relationships between the factors that we found related to the antecedent of the test for HIV infection. Prospective studies are necessary to discover whether risk beliefs, such as suspecting that the couple is HIV+, induce a greater demand for the test, or if this is explained only by the risky behaviors that are experienced by an individual.

The results cannot be extrapolated to the population level, since the sampling of the present study is non-probabilistic. In addition, the participants formed a self-selected sample, probably on the basis of their own perception of risk, as well as the knowledge and beliefs of stigma and discrimination related to HIV disease that they might have. However, these findings allow a description and approach to a topic that has been little explored in our context. 

Finally, although for this study the frequency of having an HIV test is higher than the national reports ^3^ ^-^ ^5^, especially in the traditional risk group, only 37% of them reported having a test in the last year; and in the non-traditional group of risk, this figure reached 16%, data that continue to be below the goals to strengthen the demand and offer of counseling and voluntary testing in STI-HIV/AIDS. Regarding this, it was established for the year 2015 as a target in key populations, that they undergo an HIV test in the last 12 months and know their results, an increase of 10% to 20% ^16^, compared to the baseline established for Cali, which ranged between 20% and 32% in key groups (MSM, transgender women and female sex workers). It was expected to reach a maximum coverage of 40% to 52% by 2015. These results highlight the need for more active promotion of HIV tests, accompanied by the strengthening of safe practices such as the use of condoms and other behavioral interventions that allow people with persistent risks to acquire the infection as well as the population perceived to be at lower risk, to appropriate behaviors that favor periodically testing for HIV, as indicated by national and international guidelines ^16^ ^,^ ^24^.

The results of the present study allowed us to relate the socio-demographic characteristics and behaviors/habits with a history of having an HIV test in two populations. These results allow us to know which of these characteristics could have an impact on the probability of having a previous test for HIV, and thus improve the targeting of the active search for HIV, as well as to emphasize the main changes in sexual behaviors that should be suggested to those communities within the continuous counseling for prevention.
