# Supplementary material for: Perceptions about sexual risk, HIV and HIV-testing in Cali, Colombia
Source: Colomb Med (Cali). 2018 Jun 30;49(2):139–47. doi: 10.25100/cm.v49i2.2945 (PMC6084922; doi:10.25100/cm.v49i2.2945)
Supplement: Related texts

# Research article: Percepciones sobre riesgo sexual, VIH y prueba del VIH en Cali, Colombia

## Introducción 

Hasta 2015, en Colombia se notificaron 112,110 personas seropositivas para el VIH o con sida, y 12,764 fallecidos ^1^. Según datos del Ministerio de Salud y Protección Social de Colombia, la prevalencia de la infección por el VIH en la población en general de 15 a 49 años en 2013 fue alrededor del 0.5% ^2^.

En Colombia, la toma voluntaria de la prueba para VIH está cercana al 20% en la población general ^3^, y en poblaciones de mayor riesgo para la infección osciló entre el 20% y el 54% ^4^^,^^5^; para el año 2012 se reportó que solo el 18.4 % de las personas diagnosticadas con el VIH/SIDA en Colombia habían solicitado la realización voluntaria de la prueba para el diagnóstico ^6^. Estudios de infección por VIH en poblaciones consideradas tradicionalmente de riesgo (hombres que tienen sexo con hombres (HSH), mujeres transgénero (TRANS) y mujeres trabajadoras sexuales (TS) mostraron prevalencias de VIH para la ciudad de Cali que van desde 1.7% en mujeres trabajadoras sexuales hasta 23.7% en hombres que tienen sexo con hombres ^4^^,^^7^^,^^8^. Paralelamente la estadística de antecedentes de prueba para VIH en poblaciones de mayor riesgo fue cercana al 63% ^7^^,^^8^. Reportes en población general de la ciudad de Cali en contexto de bajo nivel económico, presentaron frecuencias de VIH+ cercanas al 2% ^9^ y antecedentes de prueba de VIH que van desde 30% hasta 36 % ^9^^,^^10^.

La literatura científica sobre el tema indica que la mayoría de las personas infectadas por el VIH no se realizan la prueba antes de llegar a estadios avanzados de la infección ^11^. Sumado a esto, una gran cantidad de personas infectadas no pueden acceder al diagnóstico por diferentes razones, entre las cuales se destaca el temor al estigma y la discriminación, el costo de la prueba o la resistencia al reconocimiento de que están en riesgo ^12^^-^^15^. 

Para la ciudad de Cali, en el año 2015 se esperaba un incremento entre 10-20% ^16^ en las metas que fortalecen la demanda y la oferta de asesoría y prueba voluntaria en infecciones de transmisión sexual-VIH/SIDA frente a una línea de base establecida para la ciudad que osciló entre 20% y 32% en los grupos tradicionales de riesgo (HSH, TRANS y TS). Pese a los esfuerzos en los últimos años para mejorar el acceso a la prueba, especialmente en las personas de escasos recursos o aquellos que hacen parte de grupos tradicionales de riesgo para la infección, aún los planes y servicios de prevención para la infección son limitados o poco efectivos ^5^. 

En el contexto nacional continúa siendo escasa la información frente a la caracterización de las personas que acuden a la realización de pruebas para conocer su estado serológico de VIH, así como, el conocimiento de estadísticas de realización de pruebas de VIH. A nivel mundial y de país, existe un consenso con la importancia de hacer la prueba de VIH. Paralelamente el hecho de que la administración de la terapia antirretroviral en personas con VIH resulte en una reducción importante de la transmisión del VIH ^17^, exhorta a incrementar el diagnóstico de la infección con el propósito de mejorar la oportunidad de ingreso en la cascada de atención y tratamiento. En el presente estudio se indagaron las percepciones sobre el riesgo sexual, VIH y la prueba del VIH, al igual que los factores relacionados con el antecedente de prueba para VIH en personas pertenecientes a grupos tradicionales de riesgo para la infección por el VIH, en conjunto con parejas de personas con VIH/SIDA y personas que se identifican heterosexuales, que recibieron asesoría y prueba voluntaria para la prevención de la infección por VIH. 

## Materiales y Métodos

### Diseño

Estudio observacional de corte transversal, cuyos resultados hacen parte de un sub-análisis de datos recolectados entre los años 2012 y 2014 en Cali-Colombia, durante la implementación de una estrategia de búsqueda activa para VIH ^10^ que incluía asesoría y prueba VIH. 

### Contexto del estudio

El estudio se llevó a cabo en la ciudad de Cali, ubicada al suroeste de Colombia, con una población total aproximada de 2.3 millones de personas. Se estimó que 38% de la población de Cali está afiliada al sistema de salud subsidiado (Datos Cali en cifras 2014, Alcaldia Santiago de Cali. https://planeacion.cali.gov.co/caliencifras/Documentos%20pdf/Caliencifras2014.pdf), lo cual indica a su vez que estas personas pertenecen al nivel socioeconómico más bajo del país. 

### Población objetivo y tamaño de muestra

Participaron de manera voluntaria, por muestreo no probabilístico, personas mayores de 18 años, de ambos sexos, de estrato socioeconómico bajo. La muestra estuvo conformada por dos grupos: 1) personas de los grupos tradicionales de riesgo (HSH, TRANS, TS) y parejas de personas con VIH/SIDA (PPV); 2) personas que al momento de la entrevista se reconocían como personas heterosexuales y no pertenecientes a los grupos tradicionales de riesgo. Todos los participantes eran de bajo estrato socioeconómico 1 y 2, establecidos según clasificación de los servicios de recibos públicos para agua y energía en la ciudad (dato que fue auto reportado por los participantes). 

### Estrategia de reclutamiento

La invitación a participar se hizo a través de líderes de la comunidad pertenecientes a grupos de interés y que sirvieron como enlace para informar sobre un centro local donde los participantes podían recibir asesoramiento y acceso a la prueba VIH. Las actividades de búsqueda y desarrollo del estudio se llevaron a cabo en los centros comunitarios, lugares de trabajo sexual y en las instalaciones de un centro de atención ambulatoria para personas viviendo con VIH. La entrevista y asesoría fue realizada por personal del equipo de investigación (Médico, enfermera y trabajador social) como parte de campañas de búsqueda activa focal integral para asesoría y pruebas voluntarias de VIH (APV) acorde a las pautas nacionales ^18^.

### Recolección de la información

un cuestionario estructurado (CE) fue diseñado por el equipo de investigación para obtener información sobre características socio-demográficas, auto percepción de conocimientos sobre el riesgo y prevención del VIH, así como las variables relacionadas con comportamientos sexuales y antecedentes de infecciones de transmisión sexual (ITS), estas preguntas tuvieron en cuenta los lineamientos de los cuestionarios de las guías de prevención de VIH/SIDA del Fondo de Poblaciones de las Naciones Unidas para Colombia ^8^^,^^19^. El contenido del cuestionario fue revisado por un comité de investigación con experiencia en VIH y ajustado de acuerdo con los resultados de un estudio piloto. Una entrevista cara a cara y en paralelo con la asesoría para la prueba VIH, que permitía la aclaración inmediata de dudas o falsas creencias registradas en el cuestionario, se llevó a cabo por un profesional capacitado (enfermera, trabajador social, médico general). El CE se administró en papel y en un lugar privado. 

### Variables de estudio

#### Antecedente de prueba VIH

La variable resultado del estudio correspondió a tener una prueba previa para infección por el VIH al momento de la participación. Para evaluar el antecedente de prueba VIH, se preguntó a cada participante lo siguiente: si se habían realizado alguna vez una prueba de VIH o SIDA; posteriormente en quienes afirmaron haberse realizado una prueba antes, se indagó si se hizo la prueba por iniciativa propia, así mismo se preguntó, en qué mes y año se realizó la prueba por última vez, sin incluir las donaciones de sangre. 

#### Variables independientes

**Aspectos sociodemográficos.** A través del cuestionario se identificó la edad, sexo, nivel educativo, raza; el estrato socioeconómico, estado civil, afiliación al seguro de salud, y los ingresos mensuales.

**Uso de alcohol y drogas.** Se indagó acerca del consumo de alcohol, uso de sustancias psicoactivas (SPA). 

**Tatuajes y/o piercing.** Se indagó la realización de tatuajes y/o piercing durante el último año. 

**Comportamientos sexuales.** Se evaluaron conductas de riesgo relacionadas con el VIH, incluyendo el conocimiento sobre las vías de transmisión (que se calculó en función del porcentaje de respuestas correctas relacionadas con vías de transmisión del VIH), conocer a alguien con VIH, convivir en el momento actual o sospechar que alguna de sus parejas tiene VIH. Igualmente se indagó sobre portabilidad y uso del condón con sus parejas, prácticas de relaciones anales/orales, número de parejas sexuales (regulares, ocasionales o comerciales), sexo por dinero/beneficios, antecedente de diagnóstico de ITS por médico o profesional de salud. 

### Diagnóstico VIH

Para el diagnóstico de VIH se utilizó el algoritmo diagnóstico según la guía nacional ^20^, que se encontraba vigente durante el periodo de realización del estudio. Se tomó una primera muestra y se evaluó con prueba rápida de inmunoensayo cualitativo DoubleCheckGold™ HIV 1 & 2 (sensibilidad y especificidad del 99.9% y 99.6%-99.8%, respectivamente) ^21^. Si la primera muestra fue reactiva, se hizo una segunda muestra en papel de filtro, a la cual se le realizó una prueba de Elisa de cuarta generación, si dicha prueba era reactiva se llevó a cabo una prueba de Western Blot para la confirmación en el mismo laboratorio de referencia; si el Elisa era no reactiva, la prueba rápida de detección se consideró como “falsa positiva”.

### Análisis estadístico

Los grupos de estudio fueron descritos inicialmente mediante frecuencias y proporciones. Se realizaron comparaciones de grupos a través de la prueba Chi cuadrado Pearson y de tendencia en las variables con más de dos categorías, cuyos resultados se ilustran en las Tablas 1 y 2.

Para estimar la posible relación entre el antecedente de prueba para VIH con las características socio demográficas, conocimientos sobre el riesgo y prevención del VIH, de comportamientos sexuales y antecedentes ITS, al interior de cada grupo, se calcularon y modelaron razones de prevalencia, las cuales fueron estimadas utilizando un modelo multivariado de regresión de Poisson con varianza robusta ^22^^,^^23^. (Resultados presentados en las Tablas 3 y 4). Todos los análisis estadísticos se realizaron con Stata intercooler^®^ versión 12. El nivel de significancia utilizado para el modelo final fue 0.05. 

### Aspectos éticos

El estudio se llevó a cabo de acuerdo con los principios éticos relativos a la experimentación en humanos establecidos en Colombia por las Resoluciones 8430 de 1993 y 2378 de 2008, así como la "Declaración de Helsinki" y sus enmiendas. La participación en el estudio fue completamente voluntaria, y se obtuvo de cada participante un consentimiento informado por escrito, tanto para la asesoría y toma de prueba de VIH como para el cuestionario estructurado administrado en el estudio. Este estudio fue revisado y aprobado por el comité institucional de ética para investigación en humanos de la Corporación de Lucha Contra el Sida. 

## Resultados 

### Descriptivos

En total participaron 940 personas, La edad promedio global de los participantes fue de 28.5 ±10.9 años, y la mitad fueron hombres.

#### Análisis por grupos

Ambos grupos tuvieron una edad promedio de 28 años. La información detallada de las características generales de los participantes en cada grupo se ilustra en la Tabla 1.

**Tabla 1:** Comparación de características sociodemográficas, comportamientos y antecedentes, según grupos del estudio. Cali-Colombia, 2012-2014.

	Grupo tradicional de riesgo (HSH, Trans, TS, PPV)^*^ n= 357		Grupo no tradicional de riesgo (Hombres y mujeres heterosexuales)^*^ n= 583		*p* ^**^
	n	%	n	%	
Edad (años)					
18-25	173	49	318	54	<0.001
26-35	105	29	121	21	
36-45	58	16	75	13	
<45	21	6	69	12	
Sexo					
Masculino	255	71	216	37	<0.001
Femenino	102	29	366	63	
Descripción grupo tradicional de riesgo					
Hombres que tienen sexo con hombres	203	57			
Mujeres trans	35	10			
Mujeres trabajadoras sexuales	89	25			
Parejas de personas viviendo con VIH	30	8			
Escolaridad					
Primaria	50	14	113	19	0.005
Secundaria	207	58	351	61	
Técnico-superior	100	28	115	20	
Estado civil					
Casado -Unión libre	88	25	246	42	<0.001
Soltero-Separado-Viudo	266	75	334	58	
Afiliación en salud					
Si	281	80	526	92	<0.001
Estrato socioeconómico					
Uno (muy bajo)	165	46	328	56	0.003
Dos (bajo)	192	54	254	44	
Ingreso mensual actual (SMML)					
No tiene ingresos	118	33	309	54	<0.001
<1	122	34	166	29	
1-5	115	33	94	17	
Consumo de licor en últimos 30 días					
Si	209	63	286	50	<0.001
Relaciones sexuales cuando ha consumido licor					
Si	164	51	288	53	0.677
Consumo de sustancias psicoactivas					
Si	183	52	218	38	<0.001
En último año piercing/tatuajes					
Si	78	22	85	15	0.006
Alguna vez un MD diagnóstico ITS					
Si	70	20	80	14	0.017
Conoce alguien con VIH o muerto de SIDA					
Si	196	55	211	36	<0.001
Convive con una persona VIH/SIDA					
Si	66	19	6	1	<0.001
Sabe/sospecha que su(s) pareja(s) actual/pasada(s) tenga(n) VIH					
Si	118	33	46	8	<0.001
Pueden las personas protegerse del VIH/SIDA utilizando un condón en sus RS					
Si	305	85	515	88	0.172
Conocimiento de infección por VIH					
Adecuado	74	21	113	20	0.917
Ha tenido relaciones sexuales en últimos doce meses					
Si	344	97	523	90	<0.001
Ha tenido relaciones anales					
Si	269	77	125	23	<0.001
Ha tenido relaciones sexuales orales					
Si	303	85	324	56	<0.001
En últimos 12 meses ha recibido dinero/beneficios a cambio de RS					
Si	115	32	18	3	<0.001
Uso condón últimos 12 meses-Todas las parejas					
Sí Siempre	115	32	50	9	<0.001
Porta condones consigo					
Si	235	66	217	37	<0.001
Antecedente de test para VIH					
Si	223	62	240	41	<0.001
Tiempo desde última prueba de VIH realizada (año)					
<1	79	37	33	16	
1-3	78	37	79	38	
3-5	23	11	43	20	
>5	33	15	55	26	
Prueba realizada por iniciativa propia					
Si	180	83	158	67	<0.001
Frecuencia resultado VIH+	51	14.3	9	1.6	

*Cálculo de porcentajes columna:

**. valor *p* obtenido del test chi-2

SMML: Salario mínimo mensual legal

### Antecedente de prueba para VIH

Las personas que compartían características de los grupos tradicionales de riesgo presentaron mayor frecuencia de antecedente de prueba para VIH. Así mismo, fue mayor la frecuencia de positividad para VIH en este grupo (Tabla 1).

Considerando las diferencias encontradas entre los grupos (Tabla 1), se llevó a cabo un análisis separado de las características generales, de comportamientos y conocimientos frente al antecedente de prueba para VIH, los cuales son presentados en la Tabla 2. 

**Tabla 2:** Características generales, de comportamientos y conocimientos, evaluadas en los grupos de estudio, según antecedente de prueba para VIH, (variables con significancia <0.15, en al menos uno de los grupos). Cali-Colombia, 2012-2014.

	Grupo tradicional de riesgo (HSH, Trans, TS, PPV)*					Grupo: Grupo no tradicional de riesgo (Hombres y mujeres heterosexuales*				
	Con prueba previa (n= 223)		Sin prueba previa (n= 134)		P^**^	Con prueba previa (n= 240)		Sin prueba previa (n= 343)		P**
	n	%	n	%		n	%	n	%	
Edad (años)										
18-25	76	34	97	73	<0.0001	110	46	207	60	<0.0001
26-35	82	37	23	17		78	32	43	13	
≥36	65	29	14	10		52	22	92	27	
Sexo										
Masculino	145	65	110	82	<0.001	65	27	151	44	<0.001
Estado civil actual										
Casado -Unión libre	57	26	31	23	0.645	131	55	115	34	<0.001
Soltero	165	74	101	77		109	45	225	66	
Ingreso mensual actual (SMML)										
No tiene ingresos	50	23	68	51	<0.001	127	54	182	54	0.958
<1	87	39	35	26		70	30	96	29	
1-5	85	38	30	23		38	16	56	17	
Consumo de licor en últimos 30 días										
Si	138	66	71	57	0.097	130	55	156	47	0.055
Relaciones sexuales cuando ha consumido licor										
Si	114	57	50	41	0.006	108	47	152	48	0.846
Sustancias psicoactivas										
Si	126	58	57	44	0.013	96	40	122	36	0.327
Licor y sustancias psicoactivas										
Si	29	13	8	6	0.038	8	3	6	2	0.228
En último año piercing/tatuajes										
Si	50	23	28	21	0.746	24	10	61	18	0.01
Alguna vez un MD diagnosticó ITS										
Si	60	27	10	8	<0.001	49	61	31	39	<0.001
Conoce alguien con VIH o muerto de SIDA										
Si	139	63	57	43	<0.001	91	38	120	35	0.486
Sabe/sospecha que su(s) pareja(s) actual/pasada(s) tenga(n) VIH										
Si	81	37	37	28	0.075	19	8	27	8	0.982
Ha tenido relaciones anales										
Si	161	74	108	83	0.040	55	24	70	22	0.573
Ha tenido relaciones sexuales orales										
Si	185	84	118	91	0.093	140	62	184	59	0.433
En últimos 12 meses ha recibido dinero/beneficios a cambio de RS										
Si	92	50	23	21	<0.001	11	5	7	2	0.097
Porta condones consigo										
Si	162	74	73	55	<0.001	89	39	128	39	0.931

* Calculo de porcentajes fila;

** valor p obtenido del test chi-2

### Características generales de los participantes de estudio y su relación con antecedente de prueba previa para VIH - Análisis Multivariado

Para cada grupo, las variables con niveles de significancia menor de 0.15, fueron ingresadas en un modelo de regresión múltiple de Poisson (Tabla 3), de lo cual se obtuvo que, en ambos grupos, las características como edad y antecedente de diagnóstico médico de alguna ITS se asoció con el antecedente de prueba de VIH. En el grupo tradicional de riesgo, las prácticas de sexo comercial en los últimos doce meses y sospechar que la(s) pareja(s) actual(es)/pasada(s) tenía(n) VIH, se relacionaron con tener antecedente de prueba para VIH. En el caso de las personas no pertenecientes al grupo tradicional de riesgo, se observó que, ser mujer y convivir en pareja fueron las características más frecuentes en quienes reportaron el antecedente de realizarse una prueba de VIH (Tabla 3).

**Tabla 3:** Factores relacionados con antecedente de realización prueba para VIH, según grupos en Cali-Colombia, 2012-2014

	Antecedente realización prueba para VIH							
	Grupo: Grupo tradicional de riesgo (HSH, Trans, TS, PPV)				Grupo: Grupo no tradicional de riesgo (Hombres y mujeres heterosexuales)			
	RP	EER	p	IC 95%	RP	EER	p	IC 95%
**Edad (años)**								
18-25	Referencia				Referencia			
26-35	1.51	0.16	<0.001	1.23-1.85	1.44	0.16	0.001	1.16-1.79
<35	1.68	0.20	<0.001	1.33-2.12	0.75	0.12	0.069	0.56-1.02
**Sexo**								
Masculino	Referencia				Referencia			
Femenino	1.04	0.13	0.728	0.82-1.32	1.50	0.19	0.001	1.18-1.92
**Estado civil**								
Casado-unión libre	Referencia				Referencia			
Soltero	0.90	0.08	0.252	0.75-1.08	0.65	0.07	<0.001	0.53-0.81
**Alguna vez un MD diagnosticó ITS**								
No	Referencia				Referencia			
Si	1.25	0.11	0.007	1.06-1.48	1.72	0.18	<0.001	1.40-2.10
**Sabe/sospecha que su(s) pareja(s) actual/pasada(s) tenga(n) VIH**								
No	Referencia				Referencia			
Si	1.27	0.14	0.023	1.03-1.57	0.92	0.19	0.671	0.61-1.37
**En últimos 12 meses ha recibido dinero/beneficios a cambio de RS**								
No	Referencia				Referencia			
Si	1.52	0.17	<0.001	1.23-1.88	1.14	0.12	0.215	0.92-1.41

RP: Razón de prevalencia obtenida de modelo multivariado de Poisson;

EER: error estándar calculado por métodos robustos;

*p*: Valor *p* obtenido para cada estimador RP del de modelo multivariado de Poisson;

IC 95%: intervalo de confianza al 95% obtenido para cada estimador RP del de modelo multivariado de Poisson.

En la Tabla 4 se ilustran los resultados para ambos grupos cuando se consideró el análisis multivariado por la variable antecedente de realización de prueba VIH por iniciativa propia del participante.

Las variables asociadas con el antecedente de prueba para VIH por iniciativa propia en las personas del grupo tradicional de riesgo fueron edad mayor de 25 años, comportamientos de sexo comercial y uso de condón. Mientras que, para el grupo de personas no tradicional de riesgo, el antecedente de prueba por iniciativa propia se asoció con la categoría del intervalo de edad de 26-35 años, ser soltero y sexo comercial (Tabla 4). 

**Tabla 4:** Factores relacionados con antecedente de realización prueba para VIH por iniciativa del participante, según grupos. Cali-Colombia, 2012-2014

	Antecedente realización prueba para VIH por iniciativa del participante							
	Grupo tradicional de riesgo (HSH, Trans, TS, PPV)				Grupo no tradicional de riesgo (Hombres y mujeres heterosexuales			
	RP	EER	p	IC 95%	RP	EER	p	IC 95%
Edad (años)								
18-25	Referencia				Referencia			
26-35	1.80	0.28	<0.001	1.33-2.43	1.70	0.31	0.003	1.20-2.42
<35	2.24	0.35	<0.001	1.65-3.05	1.04	0.22	0.850	0.68-1.58
Estado civil								
Casado-unión libre	Referencia				Referencia			
Soltero	0.89	0.11	0.346	0.70-1.13	0.72	0.11	0.036	0.53-0.98
Sabe/sospecha que su(s) pareja(s) actual/pasada(s) tenga(n) VIH								
No	Referencia				Referencia			
Si	1.36	0.18	0.020	1.05-1.75	1.20	0.32	0.495	0.71-2.03
En últimos 12 meses ha recibido dinero/beneficios a cambio de								
No	Referencia				Referencia			
Si	1.31	0.16	0.031	1.02-1.67	1.62	0.38	0.038	1.03-2.56
Uso condón últimos 12 meses-Todas las parejas								
No siempre	Referencia				Referencia			
Siempre	1.40	0.16	0.003	1.12-1.74	0.96	0.32	0.909	0.50-1.8
Porta condones consigo								
No	Referencia				Referencia			
Si	1.36	0.20	0.043	1.01-1.82	1.16	0.19	0.367	0.84-1.59

RP: Razón de prevalencia obtenida de modelo multivariado de Poisson;

EER error estándar calculado por métodos robustos;

*p*: Valor *p* obtenido para cada estimador RP del de modelo multivariado de Poisson;

IC 95%: intervalo de confianza al 95% obtenido para cada estimador RP del de modelo multivariado de Poisson

## Discusión

La frecuencia de antecedente de prueba fue cercana al 50% en el total de la muestra, y se relacionó principalmente con variables como edad, sexo, estado civil, antecedentes de ITS, sospecha de una pareja con VIH, sexo comercial y uso de condón. Sin embargo, las personas del grupo tradicional de riesgo reportaron mayor frecuencia de prueba previa (62%). Los resultados sugieren que al menos una tercera parte de la población del presente estudio nunca se había hecho una prueba de VIH. En Colombia la prueba de VIH es voluntaria, no obligatoria, y siempre debe ir precedida de una asesoría, está cubierta en el Plan Obligatorio de Salud, sin embargo, en poblaciones tradicionales de riesgo dentro de lineamientos nacionales no se evidencia recomendación de frecuencia de realización de la prueba de VIH ^18^. Al respecto el centro de control y prevención de enfermedades de los Estados Unidos recomienda que todas las personas entre los 13 y 64 años se hagan la prueba del VIH al menos una vez, como parte de su atención médica de rutina y las personas con ciertos factores de riesgo se deben hacer la prueba con más frecuencia al menos una vez al año ^24^. 

La frecuencia de positividad para VIH sigue siendo alta en personas en contexto de mayor riesgo (Tabla 1), similar a la reportada para Colombia en estas poblaciones ^4^^,^^7^^,^^8^. Para el grupo de personas no tradicional de riesgo, la positividad para VIH fue superior al estimado nacional en población general ^2^. Estos resultados evidencian la necesidad de orientar estrategias diferenciadas en las poblaciones que promuevan la continuidad en los esfuerzos de prevención en VIH que se han venido adelantando en el país. 

Diferentes estudios ^3^^,^^9^^,^^25^^-^^27^ han permitido evidenciar que los factores demográficos aportan a la explicación de la realización de pruebas de VIH. En concordancia con lo anterior, el presente estudio la edad fue el factor demográfico que se relacionó con tener test previo en ambos grupos y el sexo (ser mujer) principalmente para el grupo no tradicional de riesgo, se encontró que personas mayores de 26 años del grupo tradicional de riesgo, tenían mayor probabilidad de presentar antecedente de prueba, lo cual fue similar con las personas en el rango de edad de 26 a 35 años pertenecientes al grupo no tradicional de riesgo, pero se comportó de manera inversa en los mayores de 35 años dentro del mismo grupo. Al respecto, un estudio en población general de Colombia reportó que personas con más de 65 años tienen menor probabilidad de tener una prueba de VIH ^3^. Los resultados sugieren que deben hacerse más esfuerzos diferenciados por edad y por comportamientos sexuales de riesgo que comparten las personas, como guía para optimizar la promoción de la prueba VIH. 

El reporte de antecedentes de ITS se relacionó con tener test previo en ambos grupos, observándose que entre el 61% a 86% de quienes reportaron tener diagnóstico de una ITS tenían prueba previa VIH, lo anterior sugiere la necesidad de ampliar las oportunidades de la prueba del VIH en centros de atención de ITS y en entornos clínicos similares a los que asisten tanto las personas con mayor riesgo sexual, como población adulta en general.

La sospecha o el conocimiento que la pareja actual o pasada fuera VIH+, se asoció con tener un test previo para el diagnóstico de infección por VIH, únicamente en el grupo tradicional de riesgo. Lo cual puede estar sugiriendo que el percibir este tipo de riesgo en la pareja favorece el comportamiento de realización de un test para VIH, sin embargo, la temporalidad del estudio nos limita para confirmar esta afirmación, ya que no indagamos si la prueba fue precedida por la sospecha de VIH en la pareja u otro motivo relacionado con sus comportamientos de riesgo en sí mismos, lo cual ha sido evidenciado, en personas HSH que se han realizado pruebas previas para VIH, donde se ha encontrado mayor probabilidad de reportar comportamientos de riesgo para VIH asociados a la realización de la prueba ^28^. 

La experiencia de una relación sexual por dinero se asoció con una mayor frecuencia del antecedente de la realización de la prueba VIH, para ambos grupos, principalmente cuando la prueba previa fue por iniciativa propia del participante. Diversos estudios han reportado que quienes comparten comportamientos de riesgo para infección por el VIH suelen tener más antecedente de pruebas para VIH ^28^, en algunos casos esto pudiera ser motivado por la creencia relacionada de que el trabajo de sexo comercial es de alto riesgo para la transmisión de VIH ^29^^,^^30^. Los resultados destacan la importancia de plantear estrategias de educación en personas que participan de actividades relacionadas con sexo comercial, que permitan mayor acceso a las pruebas de VIH y que sean consistente con la frecuencia de realización de misma como ha sido sugerido en este tipo de poblaciones ^24^.

La evaluación de asociaciones encontradas en ambos grupos, considerando el antecedente de realización de la prueba (Tabla 3), así como el antecedente de la realización de la prueba por iniciativa del participante (Tabla 4), incluyen variables que sugieren comportamientos de riesgo, específicamente en el grupo no tradicional de riesgo, en el cual se observó una relación entre tener una prueba por iniciativa propia y experiencia de sexo por dinero. Adicionalmente, en el grupo tradicional de riesgo al evaluar la presencia de antecedente de tener prueba por iniciativa propia, se reportó un mayor uso del condón (siempre) con todas las parejas, así como el porte de condones, esto último podría sugerir una mayor conciencia de prácticas de sexo protegido en este grupo, aunque la literatura no es clara frente al uso de condón y los antecedentes de la prueba VIH ^31^^-^^33^. Los hallazgos parecen sugerir que cuando las personas experimentan comportamientos de riesgo para una infección por VIH, se ven motivados a corregir sus comportamientos y/o a la realización de una prueba para VIH. Es necesario fomentar el acceso a la prueba a través de la demanda espontanea de la población, de la mano con estrategias que eliminen las barreras y el estigma frente a la prueba.

Las personas del grupo no tradicional de riesgo en su mayoría fueron mujeres, en este sentido, los resultados sugieren que quienes más se hacen la prueba son principalmente mujeres en edad fértil y casadas o en unión libre, esta relación podría estar mediada por la presencia o deseo de embarazo (dato no evaluado en el presente estudio); al respecto la política del país, establece que los centros de salud y hospitales con programas de control prenatal están obligados a ofrecer la prueba del VIH a toda mujer embarazada ^34^, lo cual de cierto modo puede explicar por qué el antecedente de test VIH es más frecuente en mujeres dentro de este grupo. Estos resultados sugieren la necesidad de incentivar más la promoción de la prueba de VIH en hombres que se consideran heterosexuales y no considerados dentro de los grupos tradicionales de riesgo, así como aprovechar los programas de control prenatal para sugerir la prueba VIH al hombre y no solo a la embarazada.

El diseño transversal del presente estudio limita nuestra capacidad para evaluar las relaciones temporales entre los factores que encontramos relacionados con el antecedente de prueba para la infección por el VIH. Se hacen necesarios estudios prospectivos que permiten descubrir si creencias de riesgo como sospechar que la pareja es VIH+, inducen a una mayor demanda de la prueba o si esto es explicado solo por los comportamientos de riesgo que experimenta un individuo.

Los resultados no pueden ser extrapolados a nivel de población, puesto que el muestreo del presente estudio es no probabilístico. Además, los participantes conformaron una muestra auto-seleccionada, probablemente sobre la base de su propia percepción de riesgo, así como del conocimiento y las creencias de estigma y discriminación relacionadas con la enfermedad por el VIH que pudieran tener. Sin embargo, estos hallazgos permiten una descripción y aproximación a un tema que ha sido poco explorado en nuestro contexto.

Finalmente, aunque para este estudio la frecuencia de tener una prueba de VIH es mayor a los reportes nacionales ^3^^-^^5^, sobre todo en el grupo tradicional de riesgo, solo 37% de estos reportó tener una prueba en el último año, y en el grupo no tradicional de riesgo esto llegó al 16%, datos que continúan estando por debajo de las metas para fortalecer la demanda y la oferta de asesoría y prueba voluntaria en ITS- VIH/SIDA, dentro de lo cual, para el año 2015, se estableció como meta en poblaciones clave que se someten a una prueba de VIH en los últimos 12 meses y conocen su resultados, un incremento del 10% a 20% ^16^, frente a línea de base establecida para Cali que oscilaba entre 20% y 32% en grupos claves (HSH, mujeres transgénero y mujeres trabajadoras sexuales), esperando alcanzar una cobertura máxima del 40% al 52% hacia el año 2015. Estos resultados destacan la necesidad de promoción más activa de pruebas para el VIH, acompañada del fortalecimiento de las prácticas seguras como son el uso del condón y otras intervenciones conductuales que permitan, tanto a personas con riesgos persistentes para adquirir la infección como a la población que se percibe en menor riesgo, apropiarse de comportamientos que favorezcan la realización de pruebas para detección del VIH de manera periódica, como lo indican los lineamientos nacionales e internacionales ^16^^,^^24^. 

Los resultados del presente estudio permitieron relacionar las características sociodemográficas y de comportamientos/hábitos con un antecedente de la realización de una prueba de VIH en dos poblaciones. Estos resultados permiten conocer cuáles de dichas características podrían incidir más en la probabilidad de tener un test previo para VIH y así mejorar la focalización de la búsqueda activa en VIH, lo mismo que para enfatizar sobre los principales cambios de comportamientos sexuales que se deban sugerir a dichas comunidades dentro del asesoramiento continuo para la prevención
